# A prognostic nomogram incorporating red cell distribution width for patients with intracerebral hemorrhage

**DOI:** 10.1097/MD.0000000000023557

**Published:** 2020-12-11

**Authors:** Zhe Cui, Chengwang Liu, Guozhong Sun, Liping Huang, Weiwen Zhou

**Affiliations:** a Division of Hematology and Transfusion Medicine, Department of Laboratory Medicine, Tianjin Baodi Hospital, Tianjin Baodi Affiliated Hospital of Tianjin Medical University, Baodi District, Tianjin; b Department of Oncology, the First Affiliated Hospital of Zhengzhou University, Zhengzhou, Henan Province; c Department of Emergency Medicine, Sun Yat-sen Memorial Hospital, Sun Yat-sen University, Yuexiu District, Guangzhou, China.

**Keywords:** Glasgow coma scale, intracerebral hemorrhage, nomogram, platelet distribution width, red cell distribution width

## Abstract

Intracerebral hemorrhage (ICH) is the second most common subtype of stroke with higher mortality and morbidity, and it lacks effective prognostic markers. The aim of this research is to construct newly valuable prognostic nomogram incorporating red blood cell distribution width (RDW) for ICH patients.

We retrospectively analyzed 953 adult patients with ICH. The impacts of RDW on short-term mortality and functional prognosis were calculated using Akaike information criterion (AIC), Bayesian information criteria (BIC) and the area under the curve (AUC) respectively, which could be used to compare with Glasgow coma scale (GCS) and ICH score. The independent factors of prognosis were identified by univariate and multivariate logistic regression analysis. A nomogram based on RDW for nerve functional prognosis was further constructed and validated. Its clinical value was subsequently explored utilizing decision curve analysis.

Cumulative clinical results were retrieved for 235 inpatients from Jan 2012 to June 2017. In 30-day mortality sets, GCS and ICH score had better prognostic performance than RDW (AUC: 0.929 and 0.917 vs 0.764; AIC: 124.101 and 134.188 vs 221.372; BIC: 131.021 and 141.107 vs 228.291). In 30-day functional prognosis sets, the consequences of evaluation systems were inconsistent. GCS was the best parameter for predicting outcome using AIC (262.350 vs 276.392 and 264.756) and BIC (269.269 vs 283.311 and 271.675). However, RDW was higher than GCS and ICH score considering AUC (0.784 vs 0.759 and 0.722). Age, GCS, RDW, platelet distribution width, and surgery were independent prognostic factors by multivariate logistic regression analysis, and those coefficients were used to formulate a nomogram. This nomogram can provide accurate prediction with the concordance index of 0.880 (95% CI, 0.837–0.922) higher than Harrell's concordance index of GCS system 0.759 (95% CI, 0.698–0.819) and RDW 0.784 (95% CI, 0.721–0.847). The calibration plots showed optimal consistency between bootstrap-predicted and the actual observed values of 30-day unfavorable prognosis. Decision curve analysis showed an increased net benefit for utilizing the nomogram.

High RDW values are associated with an unfavorable outcome after ICH. The established nomogram incorporating RDW should be considered for a 30-day functional prognosis.

## 1 Introduction

Intracerebral hemorrhage (ICH), the second most common pathological type of stroke, remains a cause of morbidity and mortality and is associated with significant long-term disability.^[1–4]^ Additionally, it comprises 10% to 15% of all strokes, with a global incidence rate of 24.6/100,000 and with a growing incidence related to the use of anticoagulation, antiplatelet drugs, and an aging population.^[3,4]^ Despite the ongoing efforts to improve therapeutic interventions and risk-stratification, accurately predicting the therapeutic effect of treatments and the prognosis of ICH, remains unclear. Glasgow coma scale (GCS) is a simple neurological scale that is currently used to predict the clinical outcome of ICH.^[5]^ However, several studies have demonstrated defects, including unsatisfactory prediction accuracy and omission of important prognostic factors, when using GCS.^[6,7]^ There is, therefore, an urgent need for an accurate prognostic model, which can provide guidelines for treatment and rehabilitation.

Red blood cell distribution width (RDW) is a simple and cheap hematologic parameter with multiple clinical applications.^[8]^ RDW describes the heterogeneity of circulating erythrocytes volume (anisocytosis) and is primarily used for the differential diagnosis of anemias. Increased RDW indicates a higher proportion of either large or small erythrocytes, which can be attributed to numerous metabolic disorders such as inflammatory.^[9]^ In the last decade, the number of studies investigating the correlation between RDW and human diseases has increased exponentially.^[10,11]^ RDW has also been proposed as a robust predictive marker of negative clinical outcome.^[12]^ High RDW indicates an increased incidence and all-cause mortality of cardiovascular disorders.^[13,14]^ Interestingly, in acute cerebral infarction and subarachnoid hemorrhage, RDW has been associated not only with mortality but also functional outcomes.^[15,16]^ Moreover, the inflammatory reaction has a crucial role as different RDW levels reflect the severity of ICH in patients during the initiation and progression of ICH. Altintas and his colleagues confirmed that initial RDW can provide an effective risk stratification of hematoma growth and its outcome.^[17]^

To better identify significant predictors of poor outcome, we conducted a retrospective study to assess the prognostic value of RDW. Nomogram, a new algorithm for the prognostic model, allows for simultaneous consideration of multiple predictors including the established staging system that possesses a higher power efficiency. Subsequently, we established and validated a novel nomogram algorithm incorporating significant factors and compared it with GCS using the decision curve analysis (DCA).

## 2 Method

### 2.1 Study population

The retrospective research consisted of consecutive patients admitted to the Tianjin Baodi Affiliated Hospital of Tianjin Medical University (Tianjin, China) from January 2012 to June 2017. The study was carried out in accordance with the Helsinki Declaration, based on a study protocol approved by the Ethical Committee of Tianjin Baodi Affiliated Hospital of Tianjin Medical University.

### 2.2 Inclusion and exclusion

Patients with clinical and laboratory data that met the following eligibility criteria were included:

i) 18 years of age or older,
ii) have a definite diagnosis of ICH verified by brain iconography,
iii) admitted to the stroke unit within 24 hour for ICH,
iv) primary cause of ICH (the primary reason that patients go to hospital to seek treatment is occurring ICH rather than other diseases) and
v) possessing a complete quarterly follow-up data.

The exclusion criteria were as follows:

i) not a primary cause of ICH,
ii) underlying disease affecting hematopoiesis such as hematological system disorders, chronic inflammatory, liver cirrhosis, chronic renal disease, autoimmune disorders, tumors, and other malignant diseases,
iii) use of anticoagulants and antibiotics,
iv) lack of critical clinical or follow-up data and
v) pre-stroke dependency (modified Rankin scale (mRS) score≥3).

### 2.3 Data extraction

Details were collected for all the selected patients. Demographics were obtained by a questionnaire survey, which included age, gender, and previous history of disease (e.g., diabetes, obesity, hypertension, and stroke). Clinical data on GCS, ICH volume, ICH score, and blood pressure on admission were obtained and confirmed by 2 independent clinical doctors. In addition, complete blood cell count was acquired during admission, which included hemoglobin, erythrocyte mean corpuscular volume, RDW, Neutrophil, Lymphocyte, neutrophil-to-lymphocyte rate (NLR), and platelet distribution width (PDW). Some serum biochemical parameters including creatinine, C-reactive protein and low-density lipoprotein cholesterol, then were collected. Surgery included the minimal traumatic evacuation of hematomas, traditional craniotomy, and decompression craniectomy. All the major indicators were defined by reviewing previous relative studies mentioned in the section of our instruction.

**Figure 1:**
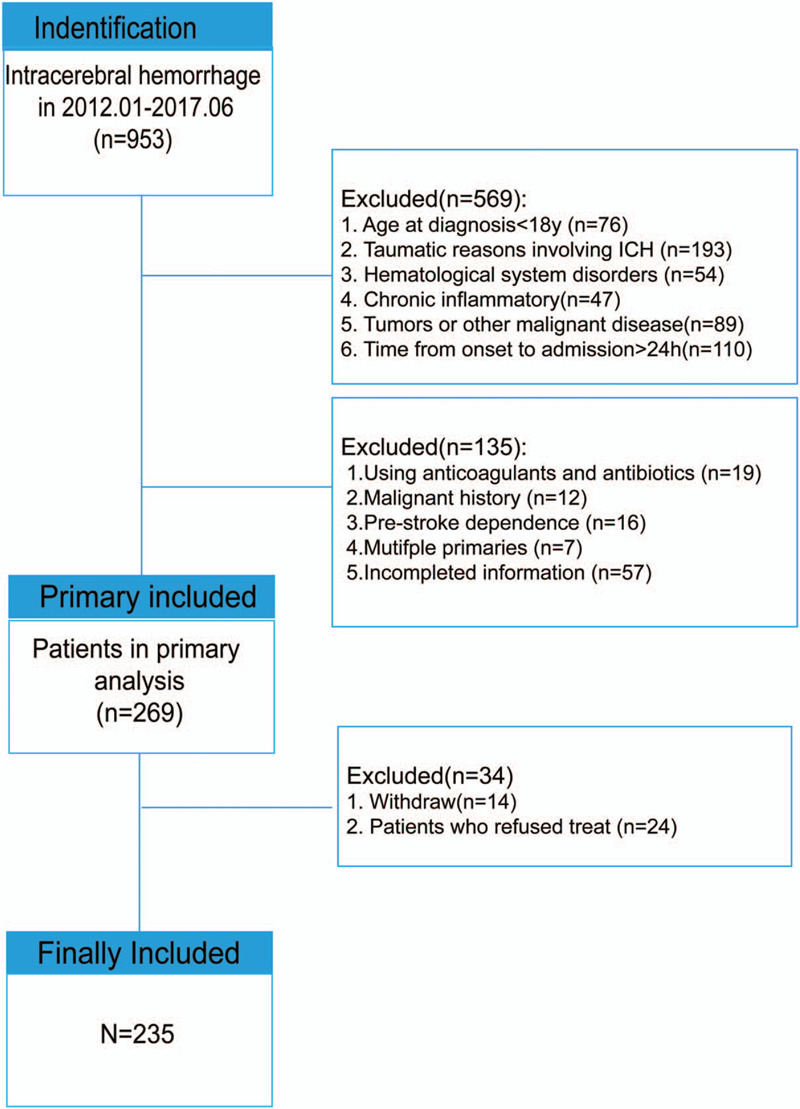
Flow diagram of selection process.

All participants were followed up for 30 days with physical and neuroimaging examinations and questionnaires regarding neurological function recovery. MRS was used as a neuro-functional evaluation scale for measuring the degree of disability or dependency with ICH.^[18]^ The 30-day mortality rate also was calculated. Details of patient selection and study development are illustrated in Figure 1. The study-enrolled patients were analyzed and divided into 2 groups according to their 30-day mortality and 30-day functional prognosis, respectively. Grouping strategies were used as follows: 30-day mortality sets (survivors vs non-survivors cohort) and 30-day functional prognosis sets (favorable cohort [mRS<3] vs unfavorable cohort [mRS≥3]).

### 2.4 Statistical analysis

We summarized continuous variables with medians and quartile ranges and used Kolmogorov–Smirnov to test for a normal distribution. Data that met a normal distribution were described as the mean ± 1 standard deviation, whereas non-normal distribution data was described by the median and quartile ranges. Student *t* test was used when normality (and homogeneity of variance) assumptions were satisfied, otherwise the equivalent to the Mann–Whitney *U* test was used. Categorical variables were expressed by frequencies/percentages, and the χ2 test or Fisher exact test was used for comparing different groups. All were conducted using SPSS version 24.0 software (IBM SPSS Statistics, Chicago, IL).

### 2.5 Prognostic performance of RDW

The receiver operating characteristic (ROC) was graphically calculated to evaluate the RDW value of the prognostic prediction and compare it with GCS and ICH score. Three methods were used to assess the comparative superiority and inferiority of various models from different aspects. The first method, Akaike information criterion (AIC) is an estimator of the relative quality of statistical models and provides means for model selection.^[19]^ The second method is the Bayesian information criteria (BIC), a useful algorithm used for the evaluation of models.^[20]^ The third method included using ROC and the area under the curve (AUC) to compare the comprehensive performance of different models. A low AIC and BIC indicate a better model fit and a high AUC indicates an effective discrimination ability for the prognostic prediction. We calculated the AIC, BIC, and AUC values of the RDW, GCS, and ICH score using the formulated statistical models and compared their prediction performance of 30-day mortality and functional prognosis in ICH patients.

### 2.6 Construction of the nomogram

As a graphical and quantitative rating prediction tool, the nomogram allows for simultaneous consideration of multiple variables including the established staging system that possesses a higher power efficiency. First, the univariate and multivariate logistic regression analyses were used to identify risk factors related to outcomes, including the 30-day mortality and functional prognosis. Variables were included in the second step of the multivariable logistic analysis regression model with backward selection (likelihood-ratio test) if they were found significantly associated with our outcomes in the first step of univariate logistic regression analysis. The above analyses were performed using SPSS version 24 (SPSS, Chicago, IL). *P* value <.05 was indicated a statistically significant difference. Second, a novel prognostic nomogram based on RDW was established for predicting the 30-day functional prognosis of ICH patients using the R software version 3.3.4 (Institute for Statistics and Mathematics, Vienna, Austria; www.r-project.org).

### 2.7 Validation of nomogram

For internal validation, 1000 bootstrap re-samples were adopted to decrease the over-fit bias. The discriminative ability of the nomogram was summarized by ROC and Harrell's concordance index (C-index). Larger the C-index and AUC, the more accurate was the prediction ability of the nomogram. The calibration curve was used to analyze the agreement between the nomogram and the ideal observation. Calibration plots on the slope of the 45-degrees line were considered as an excellent model.

### 2.8 DCA

DCA is a useful statistical tool and increasingly being used in cancer researches to determine the clinical value of prediction models. To measure the benefit of the prediction nomogram, DCA was conducted to compare the clinical usefulness of the nomogram compared to GCS and RDW. This was done by calculating the net benefits for a range of threshold probabilities. DCA was performed by R software 3.3.4. *P* < .05 was regarded as statistically significant.

## 3 Results

### 3.1 Baseline characteristics of the study

In total, 235 subjects were included in this study (median age of 64.5 years, IQR 20–90 years; 156/235 males). Fifty-two (22%) patients succumbed to ICH, and 143 (60.8%) patients were included in the functional outcome cohort within 30-day. The clinical, anamnestic, demographic, and laboratory data of the patient cohort was stratified according to the different clinical outcomes (Table 1). The median RDW on admission was 13.8 (12.4–15.2). Two types of clinical outcomes were analyzed: patents in good prognosis cohort (survivor and favorable outcome) had a lower RDW level (13.5 ± 1.3 vs 14.7 ± 1.2, *P* < .001; 13.0 ± 1.1 vs 14.2 ± 1.3, *P* < .001) relative to the bad prognosis cohort (non-survivor and unfavorable outcome), as well as for age, hematoma size, WBC, neutrophil, NLR, low-density lipoprotein cholesterol, and creatinine levels. For the prognostic score, GCS and ICH scores divided cases into groups with highly statistically significant differences in mortality and functional outcome. However, the 30-day non-survival rates and the occurrence of unfavorable neurological outcomes were significantly higher in patients who underwent surgery during admission.

**Table 1:** Comparison of clinicopathological characteristics of between 30-days mortality and 30-days functional prognosis.

		30-d mortality			30-d functional prognosis		
Characteristics	All patient (n = 235)	Survivors (n = 183)	Non-survivors (n = 52)	*P* value	Favorable outcome (n = 92)	Unfavorable outcome (n = 143)	*P* value
Age (yr)	64.6 ± 14.5	61.8 ± 13.9	74.6 ± 12.0	<.001	58.3 ± 14.2	68.7 ± 13.2	<.001
Male, n (%)	156 (66.4)	118 (64.5)	38 (73.1)	.248	58 (63.0)	98 (68.5)	.386
Comorbid diseases							
Diabetes	28 (11.9)	20 (10.9)	8 (15.4)	.382	10 (10.9)	18 (12.6)	.692
Obesity	54 (23)	48 (26.2)	6 (11.5)	.027	27 (29.3)	27 (18.9)	.063
Hypertertension	18 (7.7)	12 (6.6)	6 (11.5)	.04	2 (2.2)	16 (11.2)	.11
Stroke	131 (55.7)	105 (57.4)	26 (50)	.318	55 (59.8)	76 (53.1)	.319
BP on admission, (mmHg)							
SBP	170.1 ± 30.5	168.0 ± 29.4	177.7 ± 33.5	.042	165.4 ± 29.3	173.1 ± 13.2	.055
DBP	96.4 ± 17.9	96.5 ± 18.8	96.1 ± 14.4	.906	95.6 ± 18.0	96.9 ± 17.9	.580
MAP	121.0 ± 20.7	120.3 ± 20.9	123.4 ± 20.2	.340	118.8 ± 20.4	122.4 ± 20.9	.200
GCS score				<.001			<.001
3–6	33 (14.0)	2 (1.1)	31 (59.6)		0	33 (23.1)	
7–10	32 (13.6)	20 (10.9)	12 (23.1)		4 (4.3)	28 (19.6)	
11–15	170 (72.3)	161 (88.0)	9 (17.3)		88 (95.7)	82 (57.3)	
ICH score				<.001			<.001
0	67 (28.5)	67 (36.6)	0		41 (44.6)	26 (18.2)	
1	49 (20.9)	45 (24.6)	4 (7.7)		19 (20.7)	30 (21.0)	
2	50 (21.3)	46 (25.1)	4 (7.7)		25 (27.2)	25 (17.5)	
3	27 (11.5)	18 (9.8)	9 (17.3)		7 (7.6)	20 (14.0)	
4	25 (10.6)	6 (3.3)	19 (36.5)		0	25 (17.5)	
5	15 (6.4)	1 (0.5)	14 (26.9)		0	15 (10.5)	
6	2 (0.9)	0	2 (3.8)		0	2 (1.4)	
Hematoma size (cm^3^)	14.8 ± 17.0	11.0 ± 11.6	28.1 ± 24.7	<.001	7.7 ± 8.2	19.3 ± 19.5	<.001
Laboratory data on admission							
Hemoglobin (g/L)	136.6 ± 19.2	139.0 ± 16.7	128.2 ± 24.7	.004	140.0 ± 16.3	134.6 ± 20.6	.034
MCV (fL)	88.6 ± 6.4	88.2 ± 6.7	89.8 ± 4.7	.121	90.1 ± 4.4	87.6 ± 7.2	.001
RDW (%)	13.8 ± 1.4	13.5 ± 1.3	14.7 ± 1.2	<.001	13.0 ± 1.1	14.2 ± 1.3	<.001
WBC (10^9^/L)	11.0 ± 4.6	10.0 ± 16.7	14.8 ± 6.2	<.001	9.7 ± 3.1	11.9 ± 5.2	<.001
Neutrophil (10^9^/L)	8.5 ± 4.6	7.4 ± 3.1	12.3 ± 6.6	<.001	7.3 ± 3.1	9.3 ± 5.1	<.001
Lymphocyte (10^9^/L)	1.7 ± 1.1	1.7 ± 1.0	1.7 ± 1.2	.822	1.8 ± 0.9	1.7 ± 1.2	.529
NLR	6.8 ± 5.5	5.4 ± 3.7	11.6 ± 7.6	<.001	5.2 ± 4.0	7.85 ± 6.0	<.001
PDW (%)	12.2 ± 1.9	12.2 ± 1.9	12.5 ± 1.7	.316	11.9 ± 1.7	12.5 ± 2.0	.009
Creatinine (μmol/L)	104.1 ± 63.4	93.8 ± 0.5	140.5 ± 113.7	.005	92.4 ± 27.7	111.7 ± 77.3	.007
CRP	25.1 ± 43.6	22.3 ± 41.2	34.9 ± 50.3	.102	20.1 ± 35.8	28.3 ± 47.8	.136
LDL-C, (mmol/L)	2.8 ± 0.7	3.0 ± 0.7	2.4 ± 0.7	<.001	3.0 ± 0.7	2.7 ± 0.7	.001
Time (from onset to admission, h)	11.4 ± 8.3	11.4 ± 8.1	11.5 ± 9.2	.939	10.9 ± 8.0	11.8 ± 8.6	.468
Surgery	59 (25.1)	31 (16.9)	28 (53.8)	<.001	2 (2.2)	86 (60.1)	<.001

Bold figures indicate statistical significant *P* < .05. Surgery includes minimally traumatic evacuation of hematomas, traditional craniotomy and decompression craniectomy. BP = blood pressure, CRP = C-react protein, DBP = diastolic blood pressure, GCS = Glasgow coma scale, LDL-C = low-density lipoprotein cholesterol, MAP = mean arterial pressure, MCV = erythrocyte mean corpuscular volume, mRS = modified Rankin scale, NLR = neutrophil-to-lymphocyte rate, PDW = platelet distribution width, RDW = red blood cell distribution width, SBP = systolic blood pressure, WBC = white blood cell.

### 3.2 Comparing the prognostic impact of RDW, GCS, and ICH score

**Figure 2:**
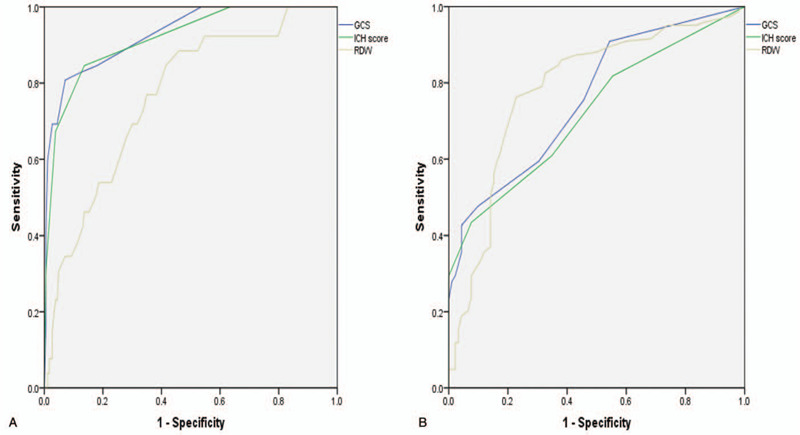
Comparison of receiver operating characteristic curves of red blood cell distribution width, Glasgow coma scale, and intracerebral hemorrhage score to predict the impact of these factors on 30-day mortality(**A**) and 30-day functional prognosis(**B**). GCS = Glasgow coma scale, RDW = red blood cell distribution width, ROC = receiver operating characteristic.

We calculated the AIC, BIC, and AUC values to compare the risk-factors of the prognostic value, as shown in Table 2 and Figure 2. In 30-day mortality sets, GCS and ICH score had a better prognostic performance than RDW (AUC: 0.929 and 0.917 vs 0.764; AIC: 124.101 and 134.188 vs 221.372; BIC: 131.021 and 141.107 vs 228.291). GCS and ICH scores better predict ICH and we, therefore, did not establish a novel prediction model. In the 30-day functional prognosis sets, the results of the evaluation systems were inconsistent. GCS was the best parameter for predicting outcome using AIC (262.350 vs 276.392 and 264.756) and BIC (269.269 vs 283.311 and 271.675). However, RDW was higher than GCS and ICH score when considering AUC (0.784 vs 0.759 and 0.722). Due to the poor efficiency of the currently utilized system, a novel predictive staging system was required.

**Table 2:** Prognostic performance of different predictive factors.

	30-d mortality			30-d functional prognosis		
	AIC	BIC	AUC	AIC	BIC	AUC
GCS	124.101	131.021	0.929	262.350	269.269	0.759
ICH score	134.188	141.107	0.917	276.392	283.311	0.722
RDW	221.372	228.291	0.764	264.756	271.675	0.784
Nomogram				207.6558	228.4133	0.880

A low Akaike information criterion and Bayesian information criteria indicate a better model fit and a high area under the curve indicates a better discrimination ability for the prognostic prediction. AIC = Akaike information criterion, AUC = area under the curve, BIC = Bayesian information criteria, GCS = Glasgow coma scale, ICH = intracerebral haemorrhage, RDW = red blood cell distribution width.

### 3.3 Constructing a nomogram for 30-day functional prognosis

**Figure 3:**
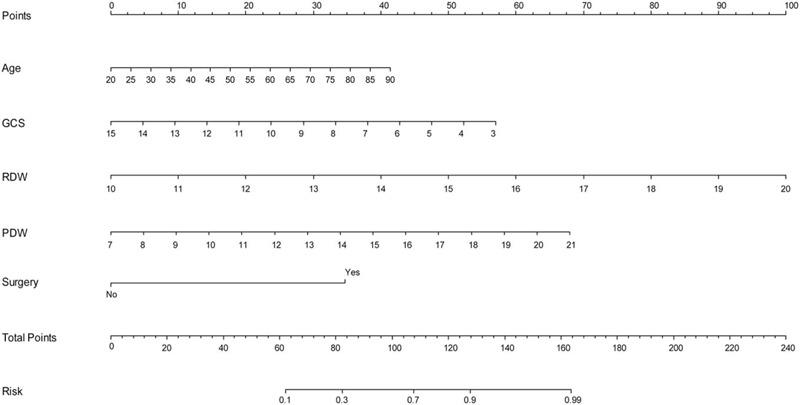
Prognostic nomogram for intracerebral hemorrhage. Nomogram including age, Glasgow coma scale, red blood cell distribution width, platelet distribution width, and surgery for predicting 30-day functional outcome after an acute intracerebral hemorrhage. The final score (i.e., total points) is calculated as the sum of the individual score of each of the 5 variables included in the nomogram. Generally, each individual involved covariate was assessed for the patient and given a point on the basis of the nomograms. GCS = Glasgow coma scale, ICH = intracerebral hemorrhage, PDW = platelet distribution width, RDW = red blood cell distribution width.

In the multivariate logistic regression analysis for the ICH score, GCS, and NLR were found significantly associated with the 30-day mortality (Table 3). Patient's age (older), lower GCS score, higher RDW or PDW, and history of surgery were related to an unfavorable prognosis (Table 4). A more accurate prognostic nomogram was proposed by integrating all afore mentioned 5 key factors (Fig. 3). RDW, GCS, and PDW were the 3 most important parameters within the nomogram. The estimated probability of 30-day unfavorable outcome can be estimated by locating and adding the scores on the total score scale. For example, the predicting probability of an unfavorable outcome for an ICH patient of 75-years, GCS=12, RDW=14.0, PDW=13, and no surgery is 72%. How did we calculate that? First, corresponding scores of these factors are located from the nomogram as 30 for “75-year-old”, 14 for “GCS=12”, 40 for “RDW=14.0”, 30 for “PDW=13” and 0 for “non-surgery”. The total score, therefore, is 114. Second, total score of 114 is equivalent to a probability of approximately 72% for an unfavorable outcome.

**Table 3:** Univariate and multivariable logistic regression analysis to identify the independent predictors of 30-days mortality of ICH.

	Univariate analysis		Multivariate analysis	
Variables	Odds ratio (95% CI)	*P*-value	Odds ratio (95% CI)	*P*-value
ICH score	0.712 (0.598, 0.860)	.007	0.673 (0.554, 0.818)	<.001
GCS	0.522 (0.392, 0.704)	.004	0.492 (0.360, 0.671)	<.001
NLR	1.189 (1.105, 1.276)	.002	1.236 (1.124,1.359)	<.001
Age	1.041 (0.816, 1.264)	.173		
Hematoma size	3.740 (0.974, 6.506)	.065		
PDW	1.212 (0.714, 1.72)	.086		
RDW	1.956 (0.792, 3.138)	.893		
WBC	1.094 (0.896, 1.292)	.072		
Neutrophil	1.180 (0.592, 1.768)	.063		
Creatinine	1.212 (0.702, 1.722)	.091		
LDL-C	2.561 (0.846, 4.276)	.115		
Surgery	19.581 (0.531, 78.613)	.358		

95% CI = 95% confidence interval, GCS = Glasgow coma scale, ICH = intracerebral haemorrhage, LDL-C = low-density lipoprotein cholesterol, NLR = neutrophil-to-lymphocyte rate, OR = odds ratio, PDW = platelet distribution width, RDW = red blood cell distribution width, WBC = white blood cell.

**Table 4:** Univariate and multivariable analysis to identify the independent predictors of functional prognosis of intracerebral haemorrhage.

	Univariate analysis		Multivariate analysis	
Variables	Odds ratio (95% CI)	*P*-value	Odds ratio (95% CI)	*P*-value
Age	1.281 (1.006, 1.556)	.009	1.038 (1.011, 1.067)	.006
GCS	0.352 (0.198, 0.560)	.032	0.174 (0.057, 0.530)	.002
RDW	1.922 (1.062, 2.782)	<.001	1.845 (1.318, 2.581)	<.001
PDW	1.189 (1.105, 1.276)	.012	1.367 (1.087, 1.718)	.007
Surery	16.131 (1.499, 61.526)	.045	12.621 (2.623, 60.728)	.002
Hematoma size	4.641 (2.833, 12.314)	.125		
ICH score	0.996 (0.896, 1.116)	.093		
NLR	1.523 (0.877,2.169)	.129		
WBC	2.094 (0.636, 4.052)	.172		
Neutrophil	2.283 (0.796, 4.170)	.136		
Creatinine	2.613 (0.825, 4.401)	.061		
LDL-C	2.352 (0.943, 3.761)	.095		

95% CI = 95% confidence interval, GCS = Glasgow coma scale, ICH = intracerebral haemorrhage, LDL-C = low-density lipoprotein cholesterol, NLR = neutrophil-to-lymphocyte rate, OR = odds ratio, PDW = platelet distribution width, RDW = red blood cell distribution width, WBC = white blood cell.

### 3.4 Validation for nomogram

**Figure 4:**
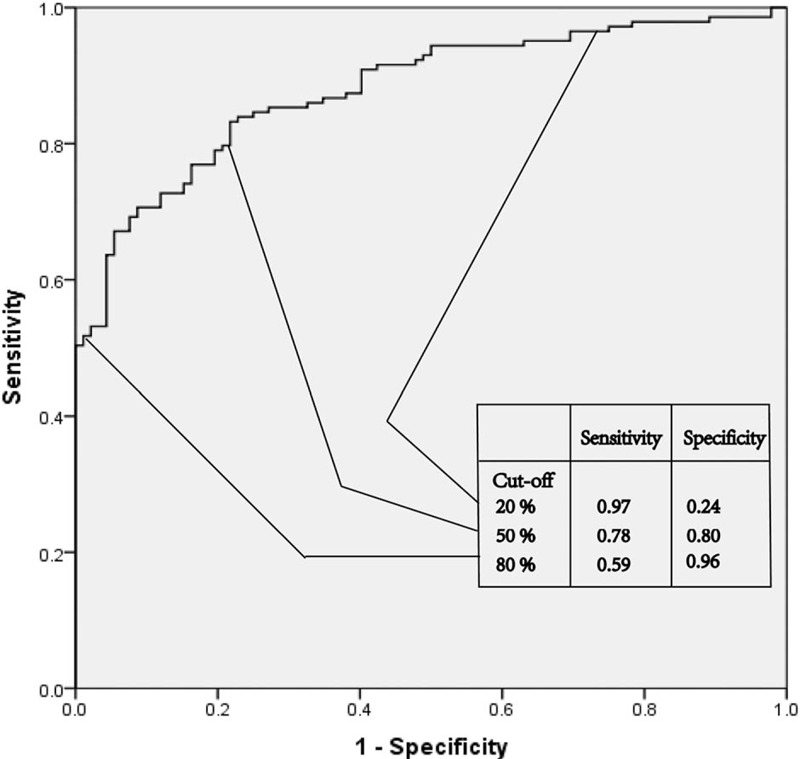
Receiver operating characteristic of the nomogram used for predicting 30-day unfavorable outcome after an acute intracerebral hemorrhage.

**Figure 5:**
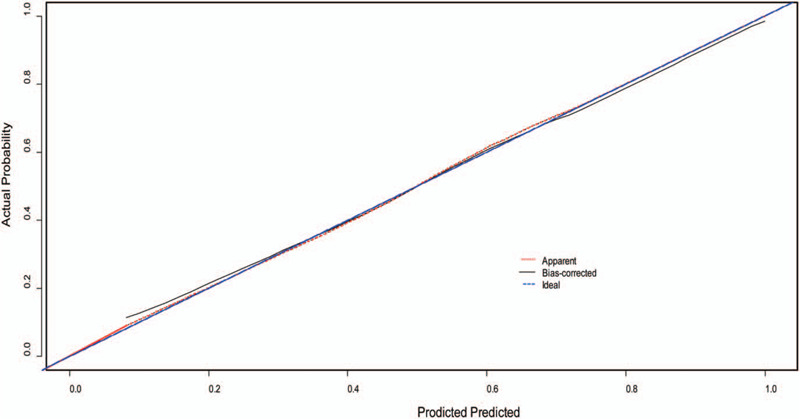
Bootstrap calibrations of nomogram. The nomogram-predicted 30-day functional is plotted on the x axis, and the actually observation is plotted on the y axis. The calibration curves predict unfavorable outcome. Blue dashed line indicates the reference line, indicating where an idea would lie. Black solid line indicates a bias-corrected calibration plot with 1000-resample bootstrapping for prediction the negative outcome at the end of the follow-up period.

Validation of the nomogram was performed using 1000 bootstrap. The C-index was 0.880 (95% CI, 0.837–0.922) higher than the C-index of GCS 0.759 (95% CI, 0.698–0.819) and RDW 0.784 (95% CI, 0.721–0.847). Furthermore, overall predictive performance was verified by means of ROC curves (Fig. 4). Altogether, this suggests that this model was reasonably accurate. The predicted survival and actual survival from the nomogram are represented by the x-axis and the y-axis, respectively. The calibration plot unraveled an adequate fit of the nomogram predicting the actual-risk of an unfavorable outcome (Fig. 5).

### 3.5 DCA of nomogram and GCS

**Figure 6:**
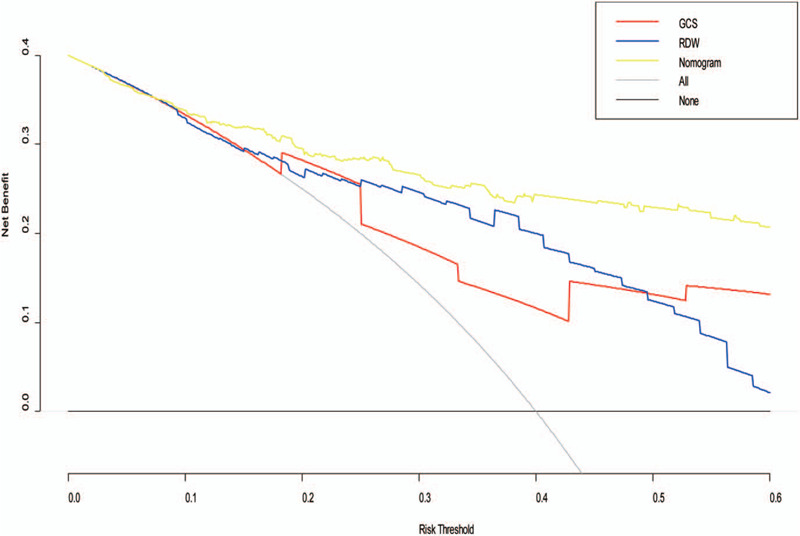
Decision curve analysis of nomograms compared with Glasgow coma scale and red blood cell distribution width. Decision curve analysis depicts the clinical net benefit in pairwise comparisons across the different models. Nomogram showed superior net benefit with a wider range of threshold probabilities compared with the Glasgow coma scale and red blood cell distribution width. DCA = decision curve analysis, GCS = Glasgow coma scale.

Clinical usefulness was evaluated as the last component of nomogram performance. DCA showed, across the entire range of threshold probability, that using the nomogram-assisted decisions to assess the 30-day unfavorable outcome provides a significant net benefit in clinical decision-making, compared to the net benefit of GCS- and RDW-assisted decisions (Fig. 6). Between the threshold probabilities of 0% to 60%, the net benefit of nomogram is clearly better than the GCS score and RDW alone.

## 4 Discussion

As the least treatable subtype of stroke, ICH has been studied intensively in order to find more powerful prognosis staging systems. RDW has gained considerable attention as its prognostic ability outperforms in several mortal-diseases.^[21,22]^ However, few studies exist concerning the risk-stratified model of ICH based on RDW. Therefore, we conducted this study using various algorithms (AIC, BIC, and AUC) in order to compare RDW with GCS and ICH score, and we subsequently identified independent factors of prognosis. A nomogram based on RDW for functional prognosis was established and validated, and its net benefit was also explored by DCA, compared with GCS score and RDW alone.

In this consecutive series of individuals with ICH, RDW was a significant prognostic factor in the univariate logistic regression analysis for short-term mortality and confirmed as an independent risk factor for functional prognosis. In the 30-day mortality sets, GSC and ICH exhibited a higher predictive accuracy. In functional prognosis sets, age, GCS, PDW, and surgery were also demonstrated as independent significant risk factors in the univariate and multivariate logistic regression analyses. Based on those predicting parameters, we constructed a nomogram for estimating the probabilities of individual 30-day functional prognosis and is an excellent predictive value compared to the GCS alone (discrimination, calibration, DCA). In addition, our research demonstrates that clinical applicability of this nomogram is feasible for ICH.

RDW, an integral part of the red blood cell automated hematology analysis without any additional costs, has built up key significant link with the adverse prognosis of life-threatening disorders including cardiovascular diseases.^[23–25]^ In 2007, Felker et al first demonstrated that RDW was a significant prognostic biomarker associated with mortality in heart failure by gathering and analyzing data from the CHARM Program and the Duke Databank.^[26]^ Several other studies on RDW have also concentrated on its prognosis prediction in cardiac-cerebral vascular diseases.^[27–29]^ A study followed 1796 patients with acute coronary syndromes (ACS) in a coronary care unit. Patients with a high RDW had a higher risk of 6-month death for ACS.^[30]^ Moreover, it was demonstrated that RDW correlates with not only short-and long-term mortality but also with functional prognosis in subarachnoid hemorrhage and ischemic stroke.^[15,16,31]^ Interestingly, a study of ICH found that an increased RDW (i.e., > 13.85) was a significant predictor of hematoma growth, relative to 3-months mRS, during an average follow-up of 2 years.^[17]^ However, there were several limitations:

i) the virtual relation of RDW for ICH prognosis was not elucidated, and
ii) the conclusion was not credible due to the small sample size (60 individuals).

In our study, we found that certain outcomes were consistent with former studies. Our work strongly supports that high RDW (AUC: 0.764 and 0.784) significantly correlates with short-team outcome (30-day mortality and unfavorable prognosis) in ICH. This also serves as an important reminder to clinicians, who should have adopted this treatment algorithm to treat those ICH patients with higher RDW level. In addition, a nomogram cooperating RDW with acceptable discrimination (C-index 0.880) and calibration was established for predicting an unfavorable outcome, and it seems to possess more power efficiency than currently utilized prognostic tools.

Despite the association between RDW and clinical outcome in ICH, the exact mechanisms are only partially understood. It still recognizes whether anisocytosis is only a participant, an onlooker, or both, in various types of vascular disease. Anisocytosis can result in an RDW change through a variety of pathogenic mechanisms, such as inflammation, oxidative stress, and nutrition deficiency.^[8]^ A high RDW may be a marker of inflammation. Elevated RDW values are correlated with sepsis, autoimmune disorders or cardiovascular disease. On 1 hand, inflammatory is frequently encountered during the development of ICH among individuals. On the other hand, inflammatory mediators may impede red cell maturation, via reduced erythropoietin production and iron bioavailability, as well as induce myelosuppression of erythroid precursors. A recent study demonstrated that a strong relationship exists between RDW and conventional inflammatory biomarkers. Allen et al (2010) elucidated that the raised RDW was closely linked to (Interleukin 6) IL-6, which strongly supports that RDW is an important marker of the inflammation.^[32]^ In addition, oxidative stress may play a role in both the process of ICH and increased RDW. Erythrocyte homeostasis and survival were affected by oxidative stress.^[33]^ More specifically, low antioxidant defenses, not only have been inversely associated with RDW but also are an independent risk factor for all-cause mortality, notably in ICH. It is well known that hematoma enlargement, hypoxia, and oxidative stress are the key factors affecting the recovery of nerve function. Moreover, Nutrition (e.g., Iron, vitamin B12, or folate) deficiency which is a common marker of impaired red cell generation,^[34]^ maybe the mechanism underlying the association between RDW and functional decline of ICH. Patel et al (year) showed that a raised RDW is positively associated with reduced erythrocyte deformability.^[35]^ Likewise, a raised RDW can inhibit endothelium-dependent nitric oxide-mediated vasodilation.^[36]^ The above 2 factors reduce the oxygen supply to damaged brain tissue and diminish the capacity for nervous system repair and recovery. Hence, anisocytosis may be an important cause of early functional decline after acute ICH.

In our study, age, GCS, RDW, PDW, and surgery were determined as independent function prognostic factors, and the ICH score had a significant association with the 30-day mortality but not the functional outcome. Currently, various prognostic tools have been proposed for the prognosis prediction after acute ICH.^[37–39]^ Age and GCS are the most consistent outcome predictors in existing forecasting models and may improve prediction efficiency through grading score, in combination with other independent outcome predictors. Parry-Jones A R et al elucidated that a model integrating age and GCS score was capable of identifying negative outcome.^[40]^ The AUC was up to 0.897, and GCS was proven as a high net benefit for threshold probabilities of 10% to 95% by DCA. Rost NS et al, by analyzing 629 consecutive patients with ICH, also reported that age and GCS were associated with functional prognosis.^[39]^ Our study found that GCS was a robust predictive factor relative to both 30-day mortality (AUC: 0.929) and functional prognosis (AUC:0.759), consistent with previous studies. Age alone was a comparatively weak predictor of mortality, but a significant prognostic factor participating in functional nomogram construction. PDW is not only a marker of platelet activation but also an important predictor of impaired reperfusion and inflammatory response, which may directly contribute to adverse functional outcome in patients with ICH. PDW is regarded as a useful prognostic factor in numerous disorders,^[36]^ especially in a stroke. Our study found similar results. Surgery is regarded as a double-edged sword and its application is controversial in ICH. We found that patients who had surgery, tended to have an unfavorable outcome. Surgery has certain risks and complications and the damage to physical function and the immune system may be led to an increase in the rate of disability and mortality. Moreover, patient with an indication for surgery may have a more serious condition. Our study suggests that surgeons should be more cautious in their understanding of surgical indications.

The nomogram in this study is innovative and has certain advantageous. First, we generated and internally validated a novel nomogram that integrated routine clinical score, laboratory variables, and treatment. The nomogram can be employed to predict early functional decline with high accuracy (AUC 0.880). Second, the 3 different statistical methods (AIC, BIC, and AUC) were used to evaluate the performance of the new model. Third, the advantage of nomogram over previous studies resides in its clinical value. Finally, our nomogram incorporates commonly accessible parameters that do not require any additional expense.

Our study was not without its limitations. First, the clinical valuation of this study may be attenuated by its retrospective nature. Given the intrinsic limitations, the effects of potential confounding on the RDW could not be assessed. Therefore, the association between RDW and ICH must be confirmed in further studies. Second, RDW is an acute-phase reactant, which may be significantly detected before ICH rather than after. However, it is difficult to access complete RDW data because of ICH unpredictability. Third, all enrolled individuals came from a single medical center. The 30-day mortality rate of the present study was 22.4%, which is similar to other oriental populations (15%–25%)^[37,41]^ and lower than western populations (31.9%, 45%).^[37,42–44]^ The reason might be attributed to racial and socioeconomic differences, suggesting multi-nation and multi-center research to eliminate the potential bias.^[45–48]^ Fourth, the data of therapeutic intervention was unavailable. As we know, medicine treatment plays a crucial role in ICH patients, notably patients without surgery. In this study, there were 34 participants in total excluded for drop-outs during follow-up. They refused therapy or withdrew in the follow-up time due to economic reasons, or other complications. As these patients accounted for a very small part of the candidates and most of their demographic characteristics matched, the influence of the exclusion on the result was minute and can nearly be ignored. Other related variables, such as “Body Mass Index”, “Diabetes or other dietary intakes”, or “Hypertension”, should also be collected and adjusted to verify the result in a larger sample size. Fifth, though we have successfully constructed a newly nomogram to help people to predict the probabilities of occurring ICH, we only performed the internal validation and lacks the external validation. We should collect another validation cohort in the future.

## 5 Conclusions

High RDW value shows an association between RDW and poor clinical outcome in patients with ICH. The established nomogram incorporating RDW should be considered for a 30-day functional prognosis.

## Acknowledgment

The authors are thankful to enrolled patients, who made the data of ICH available.

## Author contributions

**Conceptualization:** Zhe Cui, Chengwang Liu, Guozhong Sun, Liping Huang, weiwen zhou.

**Data curation:** Zhe Cui, Chengwang Liu, Guozhong Sun, Liping Huang, weiwen zhou.

**Formal analysis:** Zhe Cui, Chengwang Liu, Guozhong Sun, Liping Huang, weiwen zhou.

**Funding acquisition:** Zhe Cui, Chengwang Liu, Guozhong Sun, Liping Huang, weiwen zhou.

**Investigation:** Zhe Cui, Chengwang Liu, Guozhong Sun, Liping Huang, weiwen zhou.

**Methodology:** Zhe Cui, Chengwang Liu, Guozhong Sun, Liping Huang, weiwen zhou.

**Project administration:** Zhe Cui, Chengwang Liu, Guozhong Sun, Liping Huang, weiwen zhou.

**Resources:** Zhe Cui, Chengwang Liu, Guozhong Sun, Liping Huang, weiwen zhou.

**Software:** Zhe Cui, Chengwang Liu, Guozhong Sun, Liping Huang, weiwen zhou.

**Supervision:** Zhe Cui, Chengwang Liu, Guozhong Sun, Liping Huang, weiwen zhou.

**Validation:** Zhe Cui, Chengwang Liu, Guozhong Sun, Liping Huang, weiwen zhou.

**Visualization:** Zhe Cui, Chengwang Liu, Guozhong Sun, Liping Huang, weiwen zhou.

**Writing – original draft:** Zhe Cui, Chengwang Liu, Guozhong Sun, Liping Huang, weiwen zhou.

**Writing – review & editing:** Zhe Cui, Chengwang Liu, Guozhong Sun, Liping Huang, weiwen zhou.
